# Editorial: Using Substances to Enhance Performance: A Psychology of Neuroenhancement

**DOI:** 10.3389/fpsyg.2016.01741

**Published:** 2016-11-08

**Authors:** Wanja Wolff, Ralf Brand

**Affiliations:** ^1^Department of Sport Science, Sport Psychology, University of KonstanzKonstanz, Germany; ^2^Sport and Exercise Psychology, University of PotsdamPotsdam, Germany

**Keywords:** neuroenhancement, cognitive enhancement, doping, behavior, performance enhancement

Within the scientific community and among the general public there exists a lively debate regarding the use of drugs for the enhancement of cognitive performance. The defining feature of this type of functional substance (ab)use behavior is the *assumed functionality* a user ascribes to a chosen substance for the intended goal (e.g., Wolff and Brand, 2013; Wolff et al., 2014). According to this behavioral approach, such Neuroenhancement (NE) behavior is best understood as a goal-directed behavior that should be investigated with research that is informed by psychological theorizing. Since there is currently a lack of such research, this research topic sets out to address this gap.

An important step to advance our understanding of NE is to integrate the normative ethical debate on NE with the actual empirical evidence (Forlini and Hall). In the topics first contribution, Forlini and Hall argue that the ethical debate on the *ought* of NE (what should be done) is pursued almost entirely in isolation of what actually *is* the case. Forlini and Hall conclude that the current ethical discussion is based upon false assumptions. Namely the assumptions that NE substances have large positive effects on performance and that NE is highly prevalent. Added to these false assumptions is a lack of understanding of the psychological factors that play a role in the NE decision (Forlini and Hall).

In their comparative review of the effectiveness of pharmacological and non-pharmacological products for NE purposes, Caviola and Faber underline the first point of Forlini and Hall's analysis: Pharmacological means of performance enhancement (e.g., Methylphenidate, Modafinil) do not reliably outperform non-pharmacological ones (e.g., sleep) in terms of effectiveness. However, pharmacological means are perceived as unacceptable compared with non-pharmacological methods of performance enhancement. Faber et al.'s quantitative study indicates that the single strongest predictor of how unacceptable one evaluates NE to be is the perceived unfairness of such behavior. Thus, although no differences in effectiveness exist, pharmacological methods of NE are evaluated less positively.

The second point of Forlini and Hall's analyses referred to the implied high overall prevalence of NE. However, so far, NE prevalence has mostly been investigated in student populations. In an attempt to broaden this scope, two contributions have investigated NE prevalence outside the academic context (Dietz et al.; Sattler and Schunck). Focused on readers of a German economic newspaper, Dietz et al. found that the lifetime prevalence for lifestyle drugs NE (i.e., freely available over the counter products like Red Bull®) and illicit or prescription drugs NE was 88 and 19%, respectively. Although their sample was non-representative, these results show that NE is not merely a phenomenon among university students. Analyzing data from a representative sample of German employees, Sattler and Schunck found a considerably lower lifetime prevalence of 2.96% for prescription drugs NE. This finding aligns well with Forlini and Hall's claim that the ethical debate overstates the actual prevalence of NE.

As we have written elsewhere (Wolff et al., 2014) and in accordance with the analysis of Forlini and Hall, the NE debate lacks theory-driven research on the psychological drivers of NE. The remaining contributions have addressed this issue from different angles.

In their research perspective, Englert and Wolff carve out the relationship between NE and self-control: NE can be understood as an act of self-control that might lead to positive (performance enhancement) or negative (health issues) consequences. The postulate that NE represents a form of self-regulation is consistent with the behavioral approach to NE and is supported by the contributions of Jensen et al. and Vargo and Petróczi. In their qualitative study, Jensen et al. compared the stress and coping patterns of NE users and non-users. They found that users applied avoidant coping strategies until stress levels were unbearable. As a last resort, users then switched to the “problem focused” approach of using drugs to fulfill university requirements (Jensen et al.). Similarly, in their qualitative study, Vargo and Petróczi found that NE is used to “satisfy adaptive needs related to their work and academic demands (p. 10).” These contributions (Englert and Wolff; Jensen et al.; Vargo and Petróczi) again showcase the importance of a behavioral approach to NE that focuses on the means-end relationship represented by NE behavior. However, the contributions by Jensen et al. and Vargo and Petróczi also report that NE use seems to be more associated with a feeling of needing to catch up. This is opposed to the implicit notion of most NE definitions which suggest that NE is aimed at achieving superior performance.

In their research perspective, Zelli et al. outline a social cognitive approach that builds upon the already much more developed—and conceptually similar—field of research on doping in sports. Indeed, concepts and methodologies from this domain might well be transferable to the NE domain. For example, so called indirect measures of implicit attitudes have successfully been used in social science research on doping (Brand et al., 2014). Since NE, like doping, appears to be a socially sensitive topic and since implicit measures are less prone to faking, these measures are particularly promising for NE research as well. Part of the validation process of such measures is to understand the cortical processes that contributed to an implicit attitude score. In their contribution, Schindler and Wolff use Electroencephalography (EEG) to investigate the degree of *implicitness* that is likely to be reflected in an indirect measure of implicit attitudes toward performance enhancing substances.

The contributions of Sattler and Schunck, Brand et al., and Brand and Koch apply well-established psychological theories to NE. Sattler and Schunck's study uses the Five Factor Model of Personality and shows that NE users display lower values on conscientiousness, and higher values on neuroticism, compared with non-users. Brand et al. apply Drug Instrumentalization Theory in an attempt to broaden the view on the behavioral basis of functional substance (ab)use behaviors: Individuals can use a variety of substances (e.g., prescription drugs, illicit drugs) as instruments to achieve a variety of different goals (e.g., overcoming fatigue, facilitating social interactions). Their empirical study indicates that university students consistently use one type of drug (e.g., prescription drugs) as a means to achieve a variety of goals (as opposed to a more specialized approach of using specific drugs for specific goals). Finally, Brand and Koch use the Prototype-Willingness model to predict the willingness and intentions to use NE. In addition, they show that the theoretical links between attitudes and NE intentions was weakened when participants were given false (high) prevalence information. This finding brings us back to the point made by Forlini and Hall: A normative ethical debate that is disconnected from empirical evidence and which implies an overly high NE prevalence is problematic. Brand and Koch's results indicate that such a public discussion (building upon false premises) can, in turn, have repercussions on individuals' intentions regarding NE use.

The contributions in this research topic offer various distinctive angles on the phenomenon of NE: Engaging in ethical considerations with a focus on psychological processes will hopefully lead to better alignment between normative ethical debates and empirical evidence. Research perspectives have the potential to catalyze further theory-driven research. Qualitative approaches and research using neuroscientific methodology represent two distant points on the continuum of possible ways to understand the NE phenomenon. These different approaches can, respectively, offer either a wide, holistic perspective or a narrow, specific perspective on a phenomenon. Finally, using empirical tests based on psychological theories to differentiate users from non-users or to predict future use will hopefully prove to be a further step toward a better understanding of the psychological drivers of NE. We believe these different perspectives can mutually benefit each other and inform further, much needed research on NE behavior.

## Author contributions

WW and RB both contributed substantially to this manuscript.

### Conflict of interest statement

The authors declare that the research was conducted in the absence of any commercial or financial relationships that could be construed as a potential conflict of interest.
